# Two-dimensional biocompatible plasmonic contact lenses for color blindness correction

**DOI:** 10.1038/s41598-022-06089-8

**Published:** 2022-02-07

**Authors:** N. Roostaei, S. M. Hamidi

**Affiliations:** Magneto-Plasmonic Lab, Laser and Plasmonic Lab, Shahid Beheshti University, Tehran, Iran

**Keywords:** Biopolymers in vivo, Eye diseases, Nanoscience and technology

## Abstract

Color blindness, or color vision deficiency (CVD), is an ocular disease that suppresses the recognition of different colors. Recently, tinted glasses and lenses have been studied as hopeful devices for color blindness correction. In this study, 2D biocompatible and flexible plasmonic contact lenses were fabricated using polydimethylsiloxane (PDMS) and a low-cost, and simple design based on the soft nano-lithography method and investigated for correction of red–green (deuteranomaly) color blindness. In addition, the stability test of the fabricated plasmonic contact lenses was investigated into the phosphate buffered saline (PBS) solution and the proposed lens offers an excellent stability into the PBS solution. The plasmonic contact lens proposed herein is based on the plasmonic surface lattice resonance (SLR) phenomenon and offers a good color filter for color blindness correction. The biocompatibility, low cost, stability, and simple fabrication of these contact lenses can offer new insights for applications of color blindness correction.

## Introduction

Human color vision originates from three types of cone-shaped photoreceptors, i.e. short (S), medium (M), and long cones (L) which are responsible for perceiving blue, green, and red colors with spectral sensitivity peaks around the 430, 530, and 560 nm, respectively^1^.

Color blindness, or color vision deficiency (CVD), is an ocular disease that prevents the recognition and perception of specific colors by three photoreceptors which, in normal vision, are all present and function according to their spectral sensitivity peaks. This ocular disorder can be either acquired or congenital and is caused by the lack of or a deficiency in the cone-shaped photoreceptors^2^.

There are three different types of color blindness: dichromacy, monochromacy, and anomalous trichromacy^3^. Dichromacy occurs when one of the cone-shaped photoreceptors is completely missing and is categorized as protanopia (missing red cone cells), deuteranopia (missing green cone cells), or tritanopia (missing blue cone cells). Monochromacy is the rarest type of color blindness in which at least two cone-shaped photoreceptors are missing. Monochromat people are completely colorblind (achromatopsia) or have only blue cone-shaped photoreceptors. As the third classification, anomalous trichromacy occurs when one of the cone-shaped photoreceptors is defective. Depending on which cone-shaped photoreceptor is defective, anomalous trichromacy is divided into three categories: protanomaly (defective red cone cells), deuteranomaly (defective green cone cells), and tritanomaly (defective blue cone cells).

The most common types of color blindness are protans (protanopia and protanomaly) and deutans (deuteranopia and deuteranomaly), which are known as red-green color blindness^4^. The spectral sensitivity peak of the red cones is blue-shifted in protanomaly, while the sensitivity peak of the green cones is red-shifted in deuteranomaly. Thus, patients cannot distinguish different colors due to overlapping in the spectral sensitivity of green and red cones.

Despite a lot of useful research into a certain cure for color blindness based on different medical routes of this disease, important changes in lifestyle remain an open question. These beneficial and useful studies comprised subjects such as gene therapy^5–9^, tinted glasses^10–13^, lenses^14–17^, optical filters^18^, optoelectronic glasses, and advanced features on smartphones and computers^19–22^. Tinted glasses with color filters for color blindness correction have been widely investigated and are even commercially available^10–13,23^. These glasses block parts of the spectrum that does not affect color-blindness and are therefore not effective for improving the color perception of color-blind people^11,12^; they also have other limitations such as high cost, bulkiness, and incompatibility with other vision correction glasses.

Recently, contact lenses based on chemical dyes^24–26^, plasmonic metasurfaces^27^, and plasmonic nanoparticles^28,29^ have been investigated for color blindness correction. However, these contact lenses face challenges such as non-biocompatibility, short time usability, low stability, high cost, and the complexity of the fabrication process.

In the current study, 2D biocompatible and flexible plasmonic contact lenses based on polydimethylsiloxane (PDMS) are proposed for color blindness correction, and specific consideration is given deuteranomaly (red–green) color blindness, which is the most common type of color blindness. PDMS is a biocompatible, flexible, and transparent material which can be a good candidate for fabricating contact lenses. This nontoxic and biocompatible material has attracted many applications in fields such as biology^30–34^, medicine^35,36^, and chemistry^37^. In this work, a 2D flexible and biocompatible PDMS-based lens was successfully fabricated using a low-cost, and simple design based on the soft nano-lithography method and investigated for correction of red-green color blindness. Also, the stability test of the fabricated plasmonic contact lenses was investigated into the phosphate buffered saline (PBS) solution and the proposed lenes offers an excellent stability into the PBS solution. The biocompatibility, low cost, stability, and simple fabrication of these contact lens can offer new insights into applications for color blindness correction.

## Experimental method

**Figure 1:**
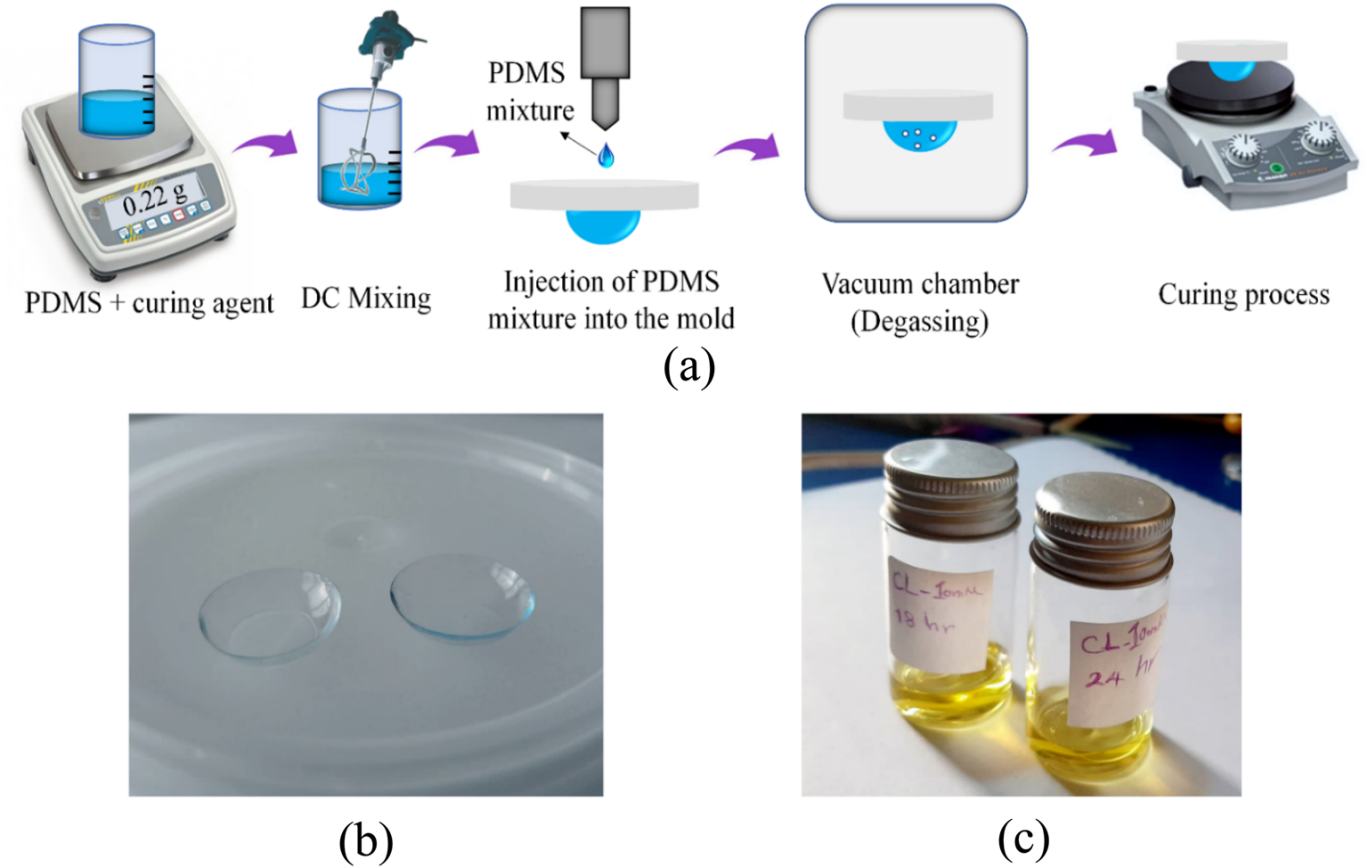
(**a**) A schematic array of the fabrication process of the proposed PDMS-based lenses, (**b**) the image of the fabricated PDMS-based lenses, and (**c**) immersing the PDMS-based lenses into HAuCl_4_·3H_2_O gold solution for different incubation times.

The PDMS-based lenses and also two dimensional biocompatible plasmonic contact lenses proposed herein were fabricated as two separate lenses with poly-dimethylsiloxane (PDMS) (SYLGARD 184 DOW CORNING). First, the proposed lenses were fabricated according to the schematic diagram shown in Fig. 1a. Firstly, PDMS was prepared by combining it with a curing agent at a weight ratio of 10:1. After mixing, these two parts by DC mixer for 5 min to achieve a homogenous mixture, which was poured onto the lens mold. For degassing, the mold was placed in a vacuum chamber for 15 min. Afterward, the sample was placed on a heater and cured with gradual increases in temperature from 50 to 100 °C over 1 h. After 24 h, the PDMS-based lens was peeled off from the mold, and thus, a biocompatible PDMS-based lens was successfully produced (Fig. 1b). In the next step, the fabricated PDMS-based lenses were immersed into 0.01 M gold solution (HAuCl_4_·3H_2_O gold chloride trihydrate) at different incubation times of 12, 18, 24, and 36 h (Fig. 1c) and then investigated for color blindness correction.

The PDMS crosslinking is originated from the reaction between silicon hydride (Si–H) groups in the curing agent and vinyl groups (Si–CHLCH2) in the monomer. After curing the PDMS, some of the Si–H groups remain which is the main factor for Au NPs production in a self-assembled method^38,39^.

**Figure 2:**
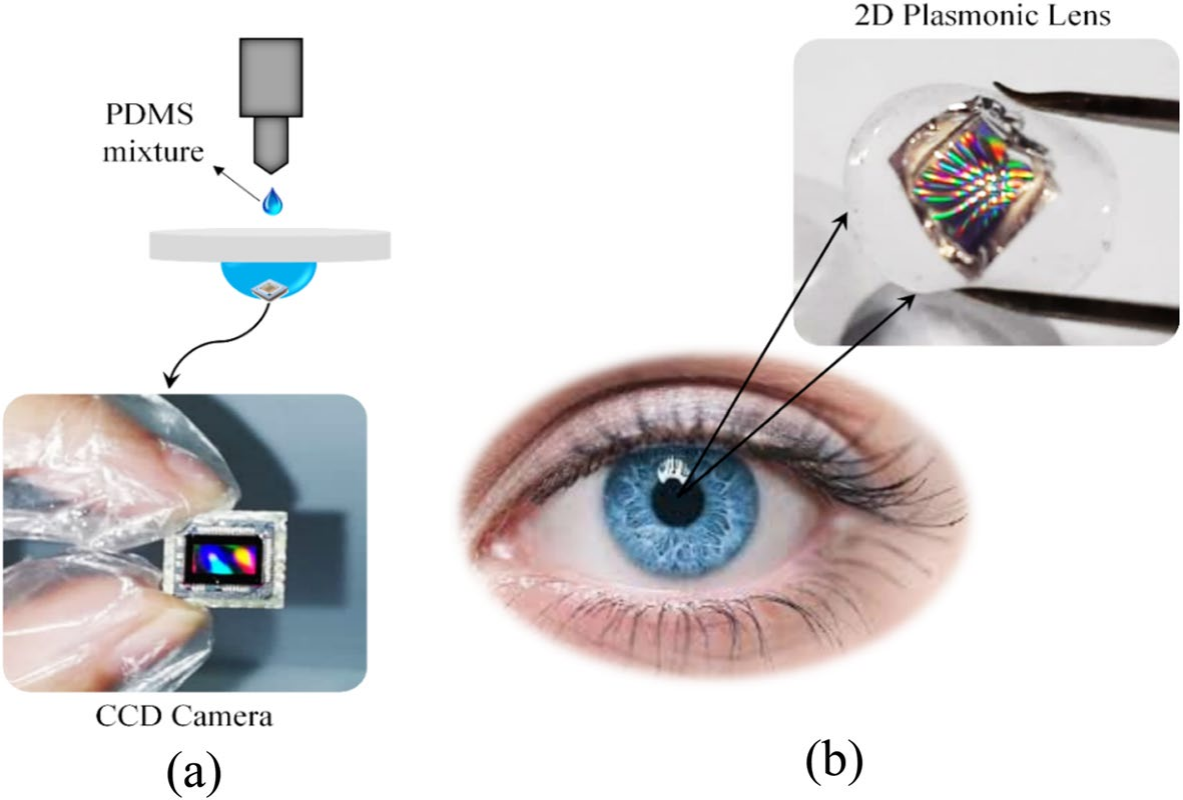
(**a**) A schematic of the fabrication process of the proposed 2D plasmonic contact lenses based on PDMS, and (**b**) the real image of the fabricated 2D flexible plasmonic contact lens.

As the second proposed structure, the 2D biocompatible plasmonic contact lenses were fabricated using the soft nano-lithography method and investigated for correction of red-green color blindness. Contact lenses should be curved due to the natural curvature of the cornea, and conventional lithography methods are only applicable for flat and planar substrates. In this research, the simple, and low-cost technique based on soft nano-lithography method was suggested to create a two-dimensional plasmonic nanostructure onto the curved surface of the lens. In this method, the charge-coupled device (CCD) of a camera was extracted and utilized as a stamp. The CCD camera had a two-dimensional periodic square pattern with a periodicity of 2.5 μm. The CCD stamp was placed into the central part of the lens mold, and a mixture of PDMS and curing agent was poured onto it (Fig. 2a). After degassing as mentioned in the previous step, the lens mold was placed on a heater and cured with gradual increases in temperature from 50 to 100 ℃ over a period of 1 h. Finally, the PDMS-based lens was separated from the mold after 24 h, and the 2D PDMS-based lens was gently peeled off of the CCD stamp. A gold layer with a thickness of 35 nm was deposited onto the patterned central part of the PDMS-based lens using the PVD technique.

In this way, a 2D flexible and biocompatible lens was successfully fabricated with a low-cost, and simple design method compared to the other costly and complex methods, such as the electron beam lithography technique. An image of the actual fabricated 2D biocompatible plasmonic contact lenses is shown in Fig. 2b.

## Simulation modeling

**Figure 3:**
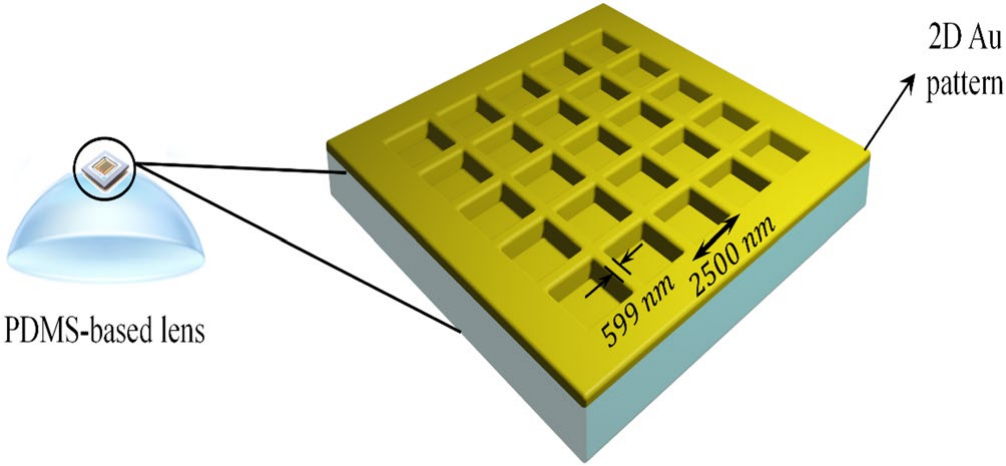
A schematic array of the simulated 2D plasmonic contact lens based on PDMS.

The fabricated two-dimensional plasmonic contact lens was simulated using the finite-difference time-domain (FDTD) method and optical electric field distribution was calculated for the proposed structure. The simulated structure consists of the 2D periodic arrays, which were arranged on the bulge part of the PDMS-based lens (Fig. 3). The lattice constant of the periodic array was considered to be 2500 nm, which was approved by scanning electron microscopy (SEM) image in Fig. 5a. The curvature radius of the simulated lens was also set at 6.9 mm, and the thickness of the thin gold layer is assumed to be 35 nm according to the experimental part.

In addition, the mesh size in the x, y and z directions was considered to be 3.5 nm and the refractive index of the PDMS and Au materials were considered from the presented data by Schneider et al. and Rakić et al. respectively^40^.

## Results and discussion

**Figure 4:**
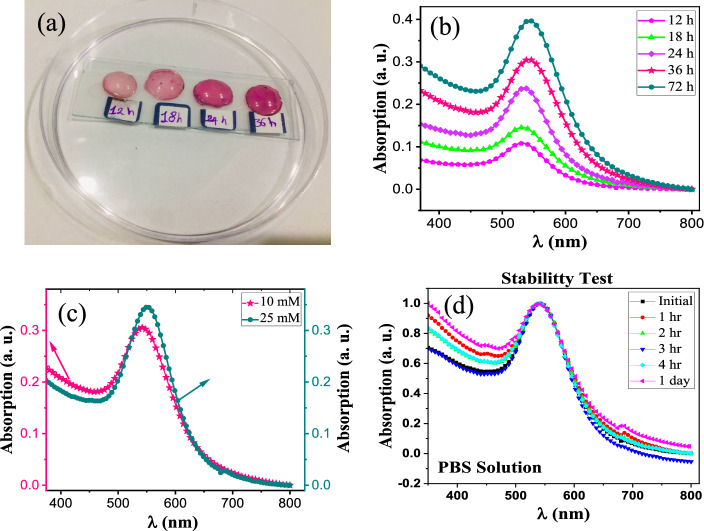
(**a**) The real image of the fabricated plasmonic PDMS-based contact lenses for different incubation times of 12, 18, 24, 36 and 72 h, (**b**) measured absorption spectra of the proposed plasmonic contact lenses with different incubation times of 12, 18, 24, 36 and 72 h, (**c**) Absorption spectra of the PDMS-based lenses which immersed into the HAuCl_4_·3H_2_O gold solutions with concentration of 10 and 25 mM for 36 h, and (**d**) stability test of the fabricated lens (with an incubation time of 36 h) into the PBS solution for different times of 1, 2, 3, 4 h and 1 day.

An image of the actual fabricated plasmonic PDMS-based contact lenses with different immersion times into HAuCl_4_·3H_2_O gold solution is shown in Fig. 4a. As shown, the color of the lenses changed with increases in immersion time, which corresponds to increases in the Au NPs content of the PDMS-based lenses. The absorption spectra of the proposed lenses with different immersion times were measured using a UV–Vis spectrometer and are shown in Fig. 4b. As can be seen, the value of the absorption peak was enhanced with increases in immersion time, which corresponds to increases in the percentage of Au NPs in the PDMS-based lenses. In addition, absorption peaks due to plasmonic resonances were observed at *λ* = 532, 533, 535, 542, 543 nm for lenses with immersion times of 12, 18, 24, 36, and 72 h, respectively. Therefore, the absorption resonance peak has a red shift of about 11 nm with increasing the immersion time from 12 to 72 h.

Also, the plasmonic resonance peak only has a red shift of 1 nm with increasing incubation time from 36 to 72 h, and no significant change in the wavelength location of the resonance response was observed. This effect indicates the stabilization of the AuNPs trapped inside the PDMS-based lens after an incubation time of 36 h; therefore, the optimum immersion time is 36 h.

The proposed plasmonic contact lens is based on the tunable localized surface plasmon resonance (LSPR) phenomenon. Plasmonic Au NPs embedded in the fabricated PDMS-based lens offer a good color filter for color blindness correction. In addition, the optical LSPR properties of gold NPs can be adjusted by controlling their morphology, including size, shape, and solvent.

The wavelength range of 540–580 nm (problematic wavelength range) must be filtered to correct deuteranomaly (red–green) color blindness, so the resonance peak must occur at the wavelength of about 560 nm. For this purpose, the concentration of the HAuCl_4_·3H_2_O gold solution was increased from 10 to 25 mM, and a PDMS-based lens was immersed in the 25 mM gold solution (HAuCl_4_·3H_2_O gold chloride trihydrate) for 36 h. For better comparison, the absorption spectra of the lenses that were immersed in the 10- and 25-mM gold solutions are given in Fig. 4c. As seen, the plasmon resonance peak has a red shift of about 11 nm with increasing the concentration of the HAuCl_4_·3H_2_O gold solution from 10 to 25 mM, and plasmonic resonance occurred at *λ* = 553 nm. In fact, the size of the Au NPs increased with increases in concentration, so the resonance wavelength had a red shift. Furthermore, the value of the absorption peak was enhanced with increases in the concentration of the HAuCl_4_·3H_2_O gold solution.

The stability test of the proposed plasmonic contact lens (with the incubation time of 36 h) into the phosphate buffered saline (PBS) solution was investigated for different times of 1, 2, 3, 4 h and 1 day (Fig. 4d). As seen, no change in the wavelength location of the absorption resonance peak was observed after immersion the lens into the PBS solution and the profile of the absorption spectra was fully preserved. Therefore, the proposed plasmonic contact lenses offer an excellent stability into the PBS solution and Au NPs are trapped inside the PDMS-based lens.

**Figure 5:**
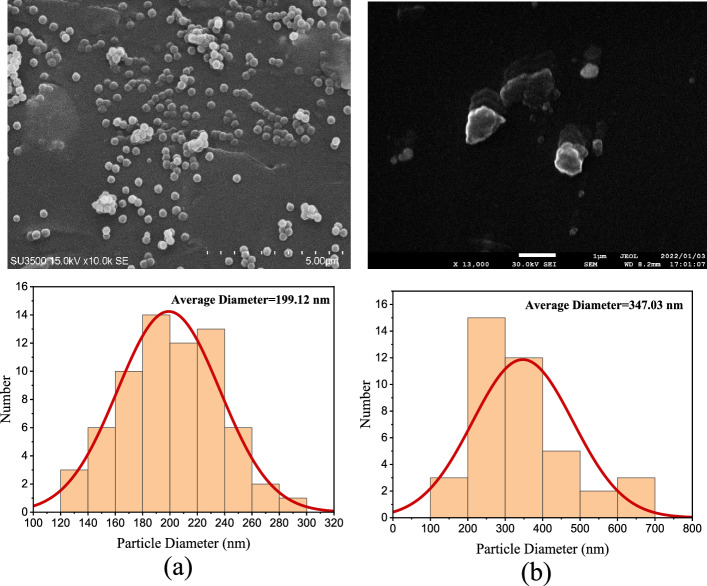
The SEM images and size distribution of the Au NPs embedded into the fabricated lenses with an incubation time of (**a**) 12 h, and (**b**) 72 h.

The SEM images of the fabricated PDMS-based lenses with an incubation time of 12 h and 72 h were recorded and shown in Fig. 5a,b, respectively. In addition, the size distribution of the Au NPs embedded into the fabricated lenses was extracted for the lenses with the incubation time of 12 h, and 72 h (Fig. 5).

The uniform distribution of the nanoparticle size with an average size of 199.12 nm was obtained for the fabricated lens with an incubation time of 12 h, while the average particle size of 347.03 nm was achieved for the lens with an incubation time of 72 h. So, the size of the nanoparticles increased with increasing time from 12 to 72 h, and the nanoparticles were agglomerated. As mentioned above, the incubation time of 36 h was considered as an optimum incubation time, and the nanoparticles were not agglomerated for this incubation time.

**Figure 6:**
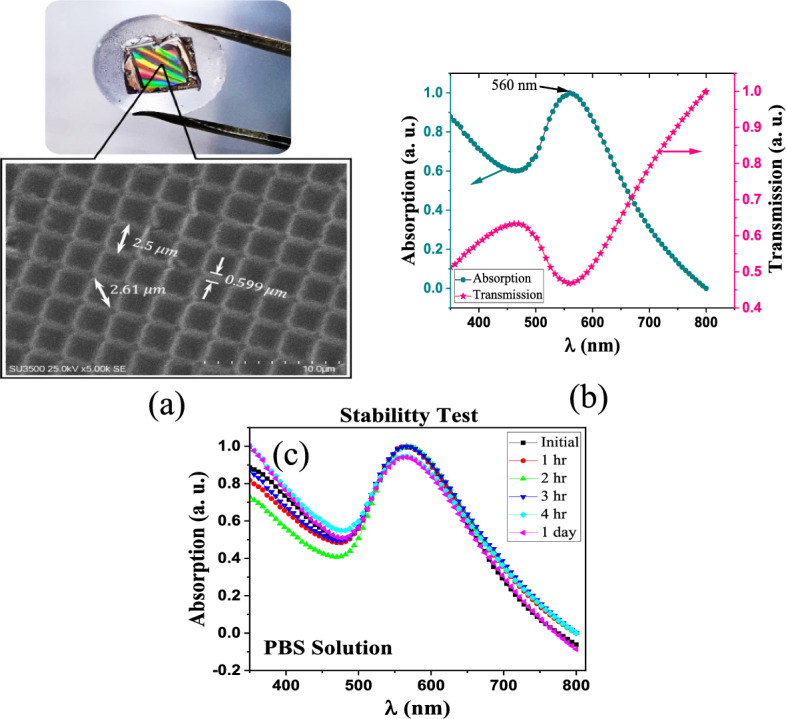
(**a**) The SEM image of the proposed 2D plasmonic PDMS-based contact lens, (**b**) measured absorption and transmission spectra of the fabricated 2D plasmonic contact lens, and (**c**) stability test of the fabricated 2D lens into the PBS solution for different times of 1, 2, 3, 4 h and 1 day.

The scanning electron microscopy (SEM) image of the fabricated 2D plasmonic contact lens is given in Fig. 6a. As can be seen, the proposed 2D plasmonic lens has a two-dimensional periodic square pattern with high resolution. Additionally, the absorption and transmission spectra of the fabricated 2D plasmonic contact lens was measured using a UV–Vis spectrometer and is shown in Fig. 6b.

Metallic nanoparticles arranged in a periodic array can exhibit extremely narrow and strong excitations known as plasmonic surface lattice resonance (SLR)^41–43^. This phenomenon is a result of the coupling between the diffracted order (DO) waves in a periodic structure and the localized surface plasmon resonances (LSPRs) coming from nanowires at the corners of each unit cell. The proposed 2D plasmonic contact lens is composed of a two-dimensional array of Au NWs and can support the sharp diffracted order (DO) waves and LSPR modes. As seen in Fig. 6b, the absorption peak occurred at *λ* = 560 nm, which corresponds to the plasmonic surface lattice resonances (SLR) caused by plasmonic 2D array of the lens, and this proposed lens offers a good color filter for the correction of deuteranomaly color blindness.

In addition, the stability test of the fabricated 2D plasmonic contact lens into the phosphate buffered saline (PBS) solution was investigated for different times of 1, 2, 3, 4 h and 1 day (Fig. 6c). As seen, no change in the wavelength location of the plasmonic resonance peak was observed after immersion the 2D lens into the PBS solution and the shape of the absorption spectra was fully preserved. Therefore, the proposed 2D lens offers an excellent stability into the PBS solution.

**Figure 7:**
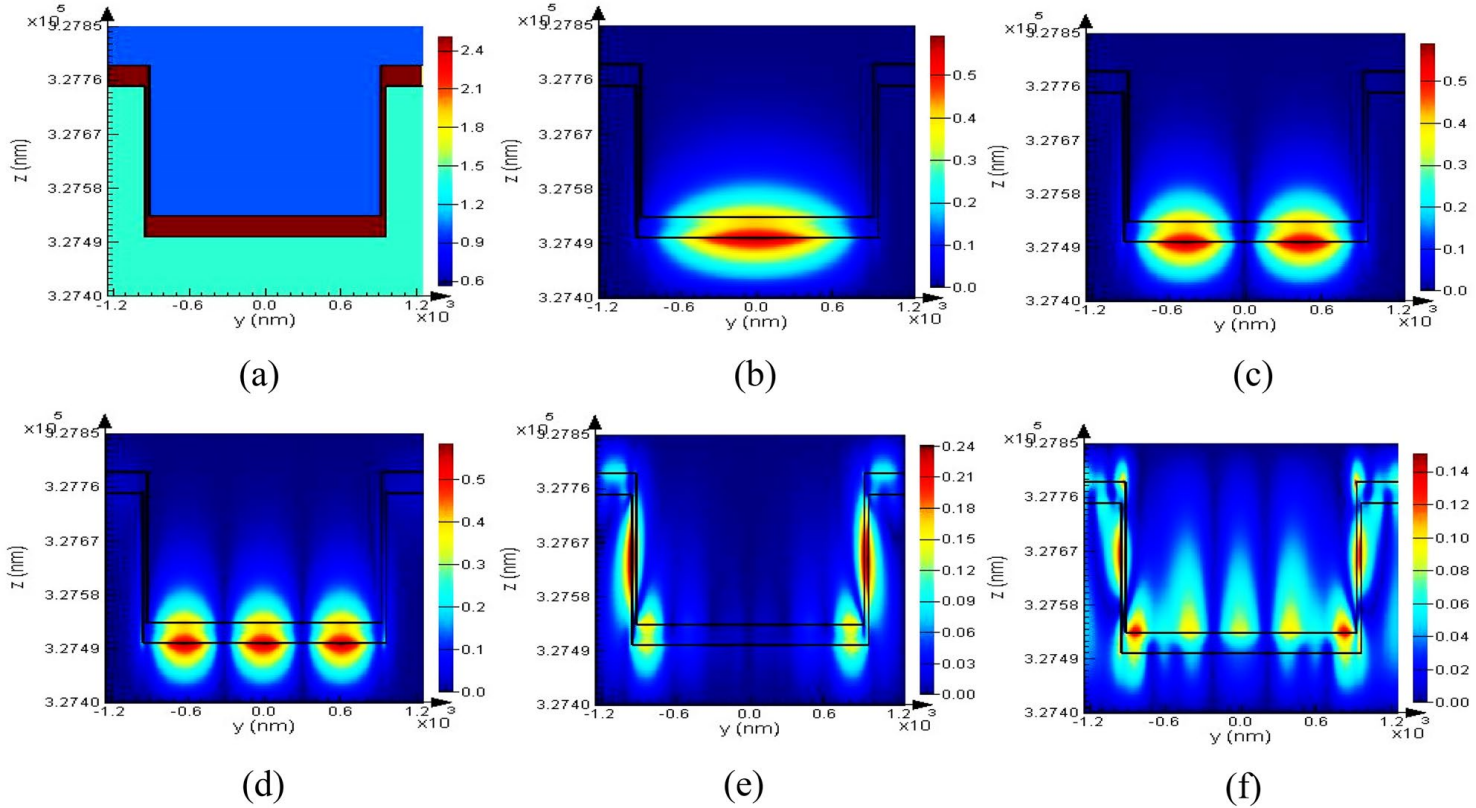
(**a**) The refractive index profile of each unit cells of the simulated structure. (**b–d**) Electric field distribution of the first three modes at *λ*_*res*_ = 560 nm, respectively, and (**e,f**) the other orders of the electric field distribution which indicate electric field localization at the sharp edges of the unit cell.

The refractive index profile of each unit cells of the simulated 2D lens and the optical electric field distribution at the absorption resonance peak (*λ*_*res*_ = 560 nm) are shown in Fig. 7a–f. As seen, electric filed localization was occurred around the gold grating at the absorption resonance peak, which is duo to surface lattice resonances (SLRs) caused by 2D plasmonic array. Furthermore, electric field enhancement was observed at the sharp edges of the unit cell (Fig. 7e,f), which indicates the strong plasmonic resonances was occurred in these regions.

## Conclusion

In this study, 2D biocompatible and flexible plasmonic contact lenses based on polydimethylsiloxane (PDMS) were fabricated with a low-cost, and simple design based on the soft nano-lithography method and investigated for correction of red–green color blindness. PDMS, a biocompatible, nontoxic, flexible, and transparent material, was used to fabricate the lens. This proposed plasmonic contact lens is based on the plasmonic surface lattice resonance (SLR) effect and can be utilized as a good color filter for the correction of deuteranomaly color blindness. The stability test of the fabricated plasmonic contact lenses was investigated into the phosphate buffered saline (PBS) solution and the proposed lenes offers an excellent stability into the PBS solution. Furthermore, the proposed lens offers excellent properties such as biocompatibility, stability, and flexibility, which can be useful for applications of color blindness correction.
