# Author Correction: Two-dimensional biocompatible plasmonic contact lenses for color blindness correction

**DOI:** 10.1038/s41598-023-36572-9

**Published:** 2023-06-09

**Authors:** N. Roostaei, S. M. Hamidi

**Affiliations:** Magneto‑Plasmonic Lab, Laser and Plasmonic Lab, Shahid Beheshti University, Tehran, Iran

Correction to: *Scientific Reports* 10.1038/s41598-022-06089-8, published online 07 February 2022

The original version of this Article contained an error in the Introduction, where it implied that tinted glasses can be used to correct color-blindness.

“While these glasses are effective for improving the color perception of color-blind people, they also have limitations such as high cost, bulkiness, and incompatibility with other vision correction glasses.”

now reads:

“These glasses block parts of the spectrum that does not affect color-blindness and are therefore not effective for improving the color perception of color-blind people^11,12^; they also have other limitations such as high cost, bulkiness, and incompatibility with other vision correction glasses.”

The original Article has been corrected.

